# Hormonal Changes Associated With Intra-Uterine Growth Restriction: Impact on the Developing Brain and Future Neurodevelopment

**DOI:** 10.3389/fendo.2019.00179

**Published:** 2019-03-26

**Authors:** Olivier Baud, Nadia Berkane

**Affiliations:** ^1^Division of Neonatology and Pediatric Intensive Care, Department of Women-Children-Teenagers, University Hospitals Geneva, Geneva, Switzerland; ^2^Inserm U1141, Sorbonne, Paris Diderot University, Paris, France; ^3^Division of Obstetrics and Gynecology, Department of Women-Children-Teenagers, University Hospitals Geneva, Geneva, Switzerland

**Keywords:** intra-uterine growth restriction, brain development, glucocorticoids, neurosteroids, insulin growth factor, thyroid hormones

## Abstract

The environment in which a fetus develops is not only important for its growth and maturation but also for its long-term postnatal health and neurodevelopment. Several hormones including glucocorticosteroids, estrogens and progesterone, insulin growth factor and thyroid hormones, carefully regulate the growth of the fetus and its metabolism during pregnancy by controlling the supply of nutrients crossing the placenta. In addition to fetal synthesis, hormones regulating fetal growth are also expressed and regulated in the placenta, and they play a key role in the vulnerability of the developing brain and its maturation. This review summarizes the current understanding and evidence regarding the involvement of hormonal dysregulation associated with intra-uterine growth restriction and its consequences on brain development.

## Fetal Growth Restriction, Brain Development, and Hormones

Fetal growth depends on several factors of maternal, fetal and placental origin, in particular genetic background, nutrients and oxygen supply to the fetus, maternal nutrition and various growth factors and hormones (1). Suboptimal fetal growth is likely to be a key factor of disruption in brain development and many neurodevelopmental disorders of motor and cognitive dysfunction have their origins in the antenatal period (2, 3). Specifically, intra-uterine growth restriction (IUGR), defined as the inability of a fetus to reach its genetically determined size is closely linked to neurodevelopmental deficits. Indeed, infants exposed to IUGR conditions are at high risk not only for neonatal death and cerebral palsy (4), but also for other neurodevelopmental morbidities including mental retardation, a wide spectrum of learning disabilities and developmental behavioral disorders associated with the onset of neuropsychiatric disorders later in life (5–7). Several of these neurodevelopmental impairments are associated not only with deleterious effect of brain undergrowth but also with IUGR-related injury of the developing brain. Magnetic resonance imaging (MRI) has clearly revealed alterations of brain development in growth-restricted infants, involving both white and gray matter (8, 9) and including altered neural circuitry identified by diffusion MRI connectomics (10, 11) that correlate with functional cognitive, motor, and psychiatric deficits later in life (12, 13).

Hormonal balance has a crucial role in fetal growth and maturation, parturition, neonatal adaptation, and brain development (14). Hormones act as maturational and nutritional signals controlling tissue development and differentiation and closely interact with the *in utero* environment. Imbalance between hormones due to placental dysfunction or antenatal chronic stress conditions don't only impair fetal maturation and growth but could also induce obstetrical, perinatal and neonatal complications including cesarean section, perinatal asphyxia, respiratory distress syndrome, abnormal glycemic regulation or inappropriate adrenal function, and hypothalamus pituitary axis (HPA) responsiveness (5).

This review recapitulates state-of-the art data based on a search in the PubMed library in English for the key words “intra-uterine growth restriction,” “hormones,” and “brain development.” The last search was done in October 2018. No restriction of year and authors were applied and review papers were used as references only for the general concepts. We identified 6 dysregulated hormones in case of IUGR, as a cause or as a result, closely related to brain development and future neurobehavioral outcomes, including glucocorticoids and oxytocin, estrogens and progesterone, insulin growth factor, and thyroid hormones.

## Fetal Growth Restriction and Chronic Exposure to Endogenous Glucocorticosteroids

### Fetal Growth Restriction, Chronic Antenatal Stress, and Glucocorticoid Exposure

Glucocorticoids (GCs) are key mediators of stress responses involved during fetal development in the regulation of fetal growth and maturation of fetal tissues and organs (15, 16). Experimental and clinical evidence indicates that increased exposure of the fetus to GCs is associated with adverse outcomes including IUGR (17), postnatal hypertension, and cardiovascular disease (18, 19), postnatal glucose intolerance, increased postnatal activity in the HPA axis (20), and interference with fetal brain development (21, 22). Conversely, placental vascular diseases leading to IUGR were found to be associated with higher plasma cortisol and lower ACTH levels compared to eutrophic fetuses (23).

Besides high concentrations of GCs observed in pregnancies complicated by IUGR, their biological effects are dependent on glucocorticoid receptors (GR), mineralocorticoid receptors, and 11beta-hydroxysteroid dehydrogenase 1/2 (11β –HSD1/2) whose expression varies over time during the antenatal period. Speirs et al. demonstrated critical periods of GC sensitivity related to changes in the expression of these molecules during antenatal development in the mouse (24). Using *in situ* hybridization they showed that GR mRNA levels were very low at embryonic day (E9.5) in the fetus but not in the placenta, and then variably rose during gestation in several tissues, including the central nervous system (CNS). In humans, both GR and the MR are highly expressed in the hippocampus from 24 weeks of gestation (25).

Fetal 11β-HSD1 mRNA expression, which could enhance GC levels locally was detected at low levels in a few brain regions, including the hippocampus only after E16.5 in mice (24). In sheep, a specific increase in the expression of 11β-HSD1 mRNA in growth-restricted fetuses in late gestation has been reported (26). In the rat, placental 11β-HSD2 is considered as a “barrier” to endogenous GCs and genetic mutations of this enzyme were found to be associated with low birthweights (27). In humans, the 11β-HSD2 gene mutation also produces IUGR which is associated with reduced placental activity of this enzyme also highly expressed in the developing brain (28). Altogether, these data, both in animals and in humans, strongly suggest that the effects of high circulating cortisol levels associated with IUGR could be potentiated by specific changes in gene expression involved in their biological response in many tissues including CNS.

### Glucocorticoids, Developing Brain, and Microglia Phenotypes

In humans, GCs regulate several developmental processes in the CNS, including hippocampal neurogenesis with variable effects on proliferation of progenitor cells, neurogenesis and astrogliogenesis in response to either low or high concentrations of cortisol (29). Low cortisol was found to increase proliferation and differentiation of progenitors into S100beta-positive astrocytes, and decrease neurogenesis. High cortisol was found to decrease proliferation and differentiation into neuronal cells without regulating astrogliogenesis. Inappropriate exposure to high levels of GCs early in pregnancy could therefore interfere with overall brain maturation. This programming effect of endogenous GCs affecting notably the HPA axis has been related to gene methylation and histone modifications associated with IUGR (30, 31) and can lead to long-lasting effects on the developing brain (32). IUGR also has sex-specific, persistent effects on hippocampal GR expression and its variants, a mechanism involved in HPA axis reprogramming, mostly in males (30).

GCs confer anti-inflammatory and immunosuppressive effects but are also able to potentiate, at high concentrations, inflammatory responses both at central and peripheral levels (33). In a model of restraint prenatal stress associated with higher levels of corticosterone investigated in juvenile and adult rats, a shift of the immune response toward a pro-inflammatory phenotype has been observed in adult rats (34). The change of GC receptor expression or function induced by IUGR could also change the microglia response toward pro-inflammatory insults associated with intensive care of growth-restricted infants, according to a multiple hit concept (35).

Extensive literature has demonstrated that chronic stress and GC exposure can impair the developing brain facing a large variety of insults, including hypoxia-ischemia, hypoglycemia, oxygen radical accumulation, all conditions potentially observed associated to IUGR (36). In preclinical models, IUGR-associated brain damage is usually associated with neuro-inflammation (5, 37–39), a key feature related to exacerbated activation of microglia, the resident macrophages of the CNS, able to sensitize the developing brain to a secondary insult (40, 41). Microglial cells can acquire distinct phenotypes in response to perinatal stimuli that allow them to either disrupt developmental processes, i.e., myelination, synaptic pruning or axonal growth, or support repair, and regeneration. These diverse roles make microglia critical modulators of brain injury and GC exposure which are able to modulate microglial phenotype both in the developing and mature brain (42–45). In the developing brain, Gómez-González et al. showed that exposure to prenatal stress alters microglia maturation leading to an imbalance between immature and ramified microglia 1 day after birth in rat (46), and increased microglial activation in the hippocampus in juvenile animals (47, 48).

### Balance Between GCs and Oxytocin

Oxytocin (OXT), an essential hormone during the perinatal period and parturition, is a neuropeptide released by the paraventricular nucleus and by the supraoptic nucleus of the hypothalamus, which is also known to be balanced against GCs. Indeed, studies carried out in rodents and in humans showed a close link between HPA axis activity and OXT release. OXT is also implicated in autism (49–51) and in the down-regulation of the central inflammatory response to injury in the mature brain (52, 53). In the developing brain, an association between IUGR, low expression of OXT and neuroinflammation, leading to defective myelination and abnormal brain function has been recently reported (54). Pharmacological treatment using carbetocin, a brain permeable long-lasting OXT receptor (OXTR) agonist, was found to be associated with a significant reduction of microglial activation and provided long-term neuroprotection. OTX also alleviated the HPA axis activation reducing GC release (55, 56), supporting the hypothesis of indirect anti-inflammatory action of OXT. These findings make OXT a promising candidate for neuroprotection, in particular in the context of IUGR.

## Sex Steroid Hormones

### Sex Steroids in Human Pregnancy and Placenta

Estradiol (E2) and progesterone (P4) are highly expressed during pregnancy (57). Sex steroids are excreted by syncitiotrophoblasts into the intervillous chambers, entering the maternal circulation, and also the fetal vessels after crossing layers of cytotrophoblasts and stromal cells. While fetal circulating E2 and P4 are mainly of placental origin, hormonal concentrations differ between maternal and fetal circulation (58), implying that some of these hormones are converted in the villi (59, 60). 17β-hydroxysteroid dehydrogenase-2 (HSD17β2) converts E2, testosterone and Δ4-androstenedione (Δ4-dione) into estrone (E1), P4, and 20α-dihydroprogesterone, respectively (61, 62). These conversions could be involved in a protective effect from excessive fetal feminization or virilization, but could also play other roles (63). In primary culture of rat hippocampal neurons, it has been shown that E2 confers protection against excitotoxic-induced cell death (64), and some E2 metabolites have various effects on fetal brain development through their receptors by promoting neurite outgrowth, myelination, and synaptogenesis (3, 65, 66) as well as neuroprotective roles (67). P4 is already used as a treatment in human adult traumatic brain injury (68).

Interestingly, recent human studies have suggested that maternal serum concentrations of E2, P4 and some of their metabolites are modified during human pregnancy complicated by preeclampsia and/or IUGR with lower placental aromatization and E2 levels and higher P4 inactivation (20α hydroxylation) (69–75).

While growing evidence demonstrates that steroidogenic enzymes are highly expressed in the CNS, below, we describe data suggesting that changes in P4 and E2 induced by IUGR could have an impact on fetal brain development and adaptation to hypoxic stress.

### Progesterone and Allopregnanolone and the Fetal Brain

Both in rodents and in humans, a large variety of brain structures (including olfactory bulb, hypothalamus, striatum, hippocampus, cerebral cortex, and cerebellum) and cell types (including glial, Purkinje, and Schwann cells) synthetize P4 (76, 77). Its 3α,5α-tetrahydroprogesterone (allopregnanolone) metabolite is mainly expressed in the cerebellum of neonatal rats (78). Progesterone receptors are expressed in rats by Purkinje cells and in the cerebellum.

Both P4 and allopregnanolone have a recognized role in neuroprotection (79). P4 has been found to induce inhibition of voltage-gated calcium channels (80) in the rat brain. Allopregnanolone found at high concentrations in maternal (81) and fetal sheep circulation (82) has been shown to have neurotrophic effects on neurons and glial cells (83, 84) in the ovine fetal brain (85).

In humans, available studies comparing controls and preeclamptic women reported conflicting results with unchanged, or higher maternal P4 concentrations (69, 74, 75). Using a reliable gas chromatography/mass spectrometry technique, no difference in maternal blood P4 and allopregnanolone concentrations was reported in women with vascular IUGR with or without preeclampsia compared to normal pregnancy (69). In contrast, in pregnant women with preexisting chronic hypertension, the development of preeclampsia was associated with higher allopregenalone concentrations (75).

In a model of IUGR developed in guinea pigs subjected to partial devascularization of the uterine horns during pregnancy, decreased allopregnanolone concentrations have been observed in fetal plasma and brain (79, 86). Moreover, in this model, despite increased expression of progesterone receptors in the brain, myelination was found to be decreased in the hippocampal region (87). Nevertheless, further studies are needed to better assess changes in P4 and its metabolite concentrations and signaling pathways involved in the adaptive response of the fetal brain to stress.

### Estradiol

In rats, estradiol (E2) is synthetized by the hippocampus (88) and cerebellum (89) and potentially by other parts of the brain with some gender differences (90). E2 effects on the brain are not yet well understood as both protecting (64, 91) and damaging effects (90, 92) have been described in primary cultures of rat hippocampal neurons. This hormone has been shown to promote axonal growth notably in cell cultures of fetal rat neurons derived from the ventromedial nucleus of the hypothalamus (93). In the oligodendroglial lineage, E2 also promotes the proliferation of immature oligodendrocytes, their differentiation into myelinating oligodendrocytes, and strongly reduces apoptotic cell death and neuro-inflammation in response to insult (94). On the other hand, as a potent regulator of the depolarizing actions of GABA, E2 can insult fetal brains subjected to hypoxic conditions by increasing the response to excessive GABA release via excess of free intracellular calcium (90).

Aromatase is a key enzyme for estrogen synthesis and several studies suggested a placental aromatase reduced activity in pregnancy with preeclampsia (70–75, 95) or with IUGR (69). As placenta is the major source of fetal estrogens, significantly lower maternal concentrations of E2, E1, or E1/Δ4-dione were reported in these pregnancies which may affect fetal estrogen levels. It can be hypothesized that during an IUGR pregnancy, a lack of fetal estrogens could disturb brain development. However, to our knowledge, no fetal or neonatal blood estrogen profile has been reported yet. Whether this abnormal placental steroidogenesis might induce changes in the steroid profile in the fetal compartment with potential brain insult remains to be determined. In addition, it is important to keep in mind that the developing brain itself has the capability to synthesize and convert sex steroids adding complexity to the interpretation of blood level data.

Despite these limitations, current evidence increasingly supports that E2, P4, and allopregnanolone play a key role in brain development and might be important modulators of brain vulnerability in the fetus with IUGR.

## Role of the Glucose-Insulin-Insulin-Like Growth Factor I (IGF-I) Axis in Placental and Fetal Growth

### Regulation of IGF Signaling in Growth Restricted Fetuses

The regulation of fetal growth depends not only on the nutrients available to the fetus but also on the regulation of Insulin-IGF/IGF binding protein 3 (IGFBP-3) axis (96, 97). The IGF factors I and II work together to control fetal growth through changes in size and function of the placenta. IGF-II is important for placental growth and development, and therefore allows more nutrients to reach the fetus. IGF-I acts as a “nutrient sensor” and finely regulates nutrient transfer across the placenta according to both the maternal environment and fetal demand. The production of IGF-I, particularly sensitive to maternal undernutrition and parental imprinting, regulates its signaling through its receptor (98). Disruption of this imprinting causes growth disorders including Beckwith–Wiedemann syndrome, associated with fetal overgrowth, and Silver-Russell syndrome, associated with IUGR (99). Several studies have shown that infants born growth restricted have lower levels of IGF-I, IGFBP-3, and insulin compared to appropriate for gestational age infants (100–102). The IGF system, IGF-I and IGF-II in particular, plays a critical role in fetal and placental growth. Disruption of the IGF-I, IGF-II, or IGF-IR gene induces IUGR, whereas disruption of IGF-IIR or overexpression of IGF-II enhances fetal growth (103).

### Placenta and IGF Signaling

Many metabolic adaptations of pregnancy are regulated by placental hormones which undergo dramatic changes during gestation including placental estrogen and progesterone (104). Placental hormone expression is supposed to interact with fetal growth through polymorphic or epigenetic regulation of placental growth hormone (PGH) and human chorionic somatomammotropin (CSH) expression could alter the expression of other critical hormones including insulin or IGF-I (105). However, definitive evidence supporting that specific placental hormones are required for normal pregnancy and fetal growth is currently lacking. It is possible that other hormones of maternal origin, such as pituitary GH and/or prolactin, might partially compensate for reduced expression of placental hormones.

### Defective IGF Signaling and Neurodevelopment

Abnormal fetal growth could also be associated with medically-induced preterm delivery (106). Low IGF-1 levels in very preterm infants and IUGR neonates were reported to be associated with high risk factor for adverse outcomes including chronic lung disease and retinopathy of prematurity. IGF-1 also plays crucial roles in the development and maturation (107) of the CNS with potent effects on cellular neuroplasticity, learning and memory, and confers neuroprotection following brain injury. IGF-1 acts at several sites to induce cellular plasticity through its receptor IGF-1R in neuronal and non-neuronal cells. IGF-1R is known to induce cellular plasticity by acting on glutamate receptors including AMPA/kaïnate-R, NMDA-R, calcium channels, and neurotransmitter release (108). Abnormal excitatory synaptic transmission observed in genetic diseases associated with behavioral disorders can be corrected by restoring SHANK3 expression or by treating neurons with IGF-1 (109). Regarding microglial activation, a major factor of brain injury, aging-related decrease in IGF-1 may contribute to the defective switch of microglia toward immunomodulatory and repair phenotype (110). During development, the IGF-1 level in cerebral spinal fluid is high, consistently with its important role in brain development, neuronal growth promotion, cellular proliferation, and differentiation (111). Finally, IGF-1 induces anti-inflammatory properties both in the developing and mature brain related to down-regulation of brain cytokine expression (112–114). Studies investigating the effects of intra-nasal IGF-1 demonstrated neuroprotection in models of LPS-induced white matter injury in the developing rat brain (115), cerebral hypoxic-ischemic injury (116, 117), and other neurodegenerative damages (118), probably through the phophatidylinositol-3 kinase/Akt pathway (119). However, the causality between low IGF-1 levels and neuroinflammation associated with IUGR remains to be confirmed.

## Thyroid Hormones

Thyroid hormones are essential for fetal brain development and maturation. Severe but also mild or subclinical neonatal hypothyroidism has been associated with neurodevelopmental impairment (120–124). However, all neonates with subclinical or mild hypothyroidism are not identified by newborn screening programs (Guthrie test). Factors associated with neonatal hypothyroidism include prematurity and IUGR (125, 126). IUGR and/or preeclampsia can be the consequences of placental insufficiency associated with overexpression of sFlt-1 (soluble fms-like tyrosine kinase-1) a soluble form of the vascular endothelial growth factor- type 1 (VEGFR-1) (127), several weeks before the beginning of maternal clinical signs (128). sFlt-1 has anti-mitogenic properties on endothelial cells (129) by trapping VEGF and placental growth factor (PlGF) leading to hypertension and proteinuria (127, 130). When IUGR occurs in human, higher sFlt-1 or sFlt-1/PlGF ratio concentrations in maternal blood have also been observed compared to the control group (131, 132). A nested case control study showed that preeclampsia predisposes to reduced maternal thyroid function (transient or permanent) (133) as others report (134). This thyroid insufficiency seems to be mediated by sFlt-1 which impairs fenestrated capillary endothelium present in endocrine glands (135). By disrupting VEGF/VEGF-R signaling in adult mice, Kamba et al. showed capillary regression in different organs, the amount of regression was dose- and organ-dependent with the highest effect in thyroid (135). Recovery of thyroid capillary density has been observed within 2 weeks after cessation of treatment. Since sFlt-1 crosses the placenta, an impact of fetal thyroid function could also be suspected with risk of subsequent neurodevelopmental impairment. Cord blood sFlt1 concentrations have been found to be inversely correlated to birthweight (136) and free T4 and positively correlated with thyroid stimulating hormone (TSH) (137). However, conflicting findings have been reported regarding the effect of IUGR on fetal serum concentration of thyroid hormones. In a series of 49 growth-restricted fetuses who had cordocentesis during pregnancy, higher concentrations of TSH and lower concentrations of free T4 have been found compared to fetuses with appropriate growth for gestational age, and changes in TSH concentrations were correlated to fetal hypoxia and academia (138). In contrast, others reported unchanged or low TSH levels in IUGR cord blood compared to controls (139–142). Variability in fetal exposure to sFlt1 could be involved in TSH regulation and may partly explain these conflicting findings.

In summary, IUGR leads to neuropathological consequences for the developing brain with heterogeneous features and long-term neurocognitive and behavioral consequences (5, 143). Many factors contribute to the vulnerability of the developing brain, including age of delivery, severity of *in utero* compromise, co-morbidities occurring during the perinatal period, complications associated with medically-induced preterm delivery. Changes in several hormones strongly involved in the regulation of brain development and maturation are likely to play a key role. More research is needed to better understand these crosstalks.

## Author Contributions

OB and NB did the literature review and collectively analyzed articles selected in this review paper. OB wrote paragraphs related to introduction, glucocorticoids, oxytocin, and IGF1. NB wrote paragraphs related to sex steroids and thyroid hormones.

### Conflict of Interest Statement

The authors declare that the research was conducted in the absence of any commercial or financial relationships that could be construed as a potential conflict of interest.
